# Tuberculosis and Nontuberculous Mycobacterial Infections in Patients with Spondyloarthritis: A Population-Based Study

**DOI:** 10.3390/medicina60040579

**Published:** 2024-03-31

**Authors:** Jiyoul Yang, Hyun-a Jang, Hyunjeong Cho, Yo Han Im, Ji Hyoun Kim

**Affiliations:** 1 Division of Pulmonary and Critical Care Medicine, Department of Internal Medicine, Chungbuk National University Hospital, Cheongju 28644, Republic of Korea; 2 Department of Environmental Science, Baylor University, Waco, TX 76798, USA; jhyunaa94@gmail.com; 3 Division of Nephrology, Department of Internal Medicine, Chungbuk National University Hospital, Cheongju 28644, Republic of Korea; 4 Division of Rheumatology, Department of Internal Medicine, Chungbuk National University Hospital, Cheongju 28644, Republic of Korea

**Keywords:** ankylosing spondylitis, psoriatic arthritis, Mycobacterium tuberculosis, nontuberculous mycobacterial infection, biologics

## Abstract

*Background and Objectives*: Tuberculosis is caused by Mycobacterium tuberculosis (MTB), while nontuberculous mycobacteria (NTM) encompass a group of mycobacterial species that are distinct from the MTB complex and leprae. Spondyloarthritis (SpA) is a group of chronic inflammatory diseases with shared clinical characteristics and is treated with biological agents; however, their use may elevate the risk of MTB and NTM infections. This study aimed to compare the incidence and risk of MTB and NTM infections in patients with SpA, including ankylosing spondylitis (AS) and psoriatic arthritis (PsA), using a population-based approach. *Materials and Methods*: This study included 2333 patients with SpA and 9332 age- and sex-matched controls from the Korea National Health Insurance Service-National Sample Cohort database from 2002 to 2019. The patients were identified using the International Classification of Diseases-10 codes for AS, PsA, MTB, and NTM. *Results*: The results showed that a negligible percentage of patients with SpA developed NTM (0.002%) and MTB (0.016%), with no significant difference in the incidence rate ratio (IRR) compared to controls. Among patients with SpA treated with biologics, the IRRs for NTM and MTB were 5.66 and 3.069, respectively; however, these were not statistically significant. No cases of NTM or MTB infection were reported in female patients with SpA treated with biologics. In both the SpA patient group and the control group, the incidence of MTB was higher in individuals over 60 years old compared to those under 60 years old. Cox proportional hazard analysis revealed a significant adjusted hazard ratio of 1.479 for MTB in patients with SpA after adjusting for age, sex, smoking history, insurance level, and comorbidities. However, this significance was not maintained when biological therapy was further adjusted. *Conclusions*: Our study indicated that the risks of NTM and MTB infection are not elevated in patients with SpA. Although biological use may potentially increase the risk of MTB infection, it does not lead to a significant increase in incidence rates. Proactive screening for latent tuberculosis and adequate prophylaxis using biologics can effectively manage the risk of NTM and MTB infections.

## 1. Introduction

Tuberculosis (TB) remains a major public health burden and a leading cause of deaths worldwide [1]. It is caused by Mycobacterium TB (MTB) and propagates among individuals via airborne transmission [2]. The worldwide incidence of TB in 2018 increased by 11% from 2008 [3].

Nontuberculous mycobacteria (NTM) encompass a group of mycobacterial species that are distinct from the MTB complex and leprae. Over 160 species have been identified in this category [4,5]. NTM is ubiquitous in the environment, rendering the epidemiological characterization of NTM pulmonary disease challenging [6]. In recent decades, the global prevalence of NTM infections has increased [7]. A study conducted in South Korea revealed an increased prevalence of MTB among younger individuals [8], wherein NTM infections occurred predominantly in women and older adults [9].

Spondyloarthritis (SpA) is a group of chronic inflammatory diseases with shared clinical characteristics and genetic linkages, such as the presence of human leukocyte antigen B27. These disorders typically manifest as a propensity for new bone formation, enthesitis, and frequent involvement of the axial skeleton, including the sacroiliac and peripheral joints. SpA can be categorized into two primary subtypes, namely predominantly axial and predominantly peripheral [10,11]. Ankylosing spondylitis (AS) is an axial variant of SpA characterized by radiological alterations in the sacroiliac joints. The SpA group also includes patients with psoriatic arthritis (PsA), which is arthritis associated with inflammatory bowel disease, and undifferentiated arthritis [10,11]. PsA is a heterogeneous chronic immune-mediated inflammatory joint disease. This condition is characterized by several distinct features, including arthritis affecting both the spine and limbs, inflammation at sites where tendons or ligaments attach to the bone (enthesitis), swelling of the fingers or toes (dactylitis), and associated skin and nail changes [12]. Nonsteroidal anti-inflammatory drugs (NSAIDs) are the primary therapeutic agents for AS and SpA; however, when NSAIDs fail to adequately control symptoms, the use of biological agents, such as tumor necrosis factor (TNF) alpha inhibitors and interleukin (IL)-17 inhibitors, may be considered. These biologics specifically target key cytokines that play crucial roles in AS [13].

In other words, SpA is a chronic inflammatory disease, and the use of immunomodulating agents such as conventional disease-modifying antirheumatic drugs (DMARDs) or biologic DMARDs may lead to decreased immunity and increased susceptibility to infection. Current treatment guidelines advise testing for latent TB prior to initiating biologic agents, and if the results are positive, initiating prophylactic treatment. Given that TB is an opportunistic infection more likely to occur in immunocompromised individuals, we conducted research with a particular interest in this aspect. These hypotheses posit an elevated incidence of MTB or NTM infections in patients with chronic inflammatory diseases, such as SpA, compared with the general population. Furthermore, this suggests potential variations in infection rates based on age or sex, influenced by the pharmacological agents employed in SpA treatment. This study aimed to compare the incidence and risk of MTB and NTM infections among patients with AS and PsA and the general population using a population-based methodology. Additionally, it endeavored to assess the impact of exposure to various treatments, including NSAIDs and biologics, on patients with AS and PsA.

## 2. Materials and Methods

### 2.1. Data Sources

In this retrospective cohort study, using the 2002–2019 Korea National Health Insurance Service-National Sample Cohort (KNHIS-NCS) database, we conducted a population-based study for patients diagnosed with AS and PsA. The KNHIS is a universal and mandatory health insurance service that provides extensive medical care coverage to approximately 97% of the Korean population. The KNHIS-NSC dataset consists of 1 million individuals selected by systematic stratified random sampling and includes extensive information about diagnoses, such as International Classification of Diseases Tenth Revision (ICD-10) diagnosis codes, demographics, health insurance type, death records, medical services, cost from all clinics, and medical utilization (https://nhiss.nhis.or.kr/bd/af/bdafa001lv.do, accessed on 24 May 2022). However, this site is accessible only to those who have obtained qualifications owing to information protection issues. This study was approved by the Institutional Review Board of the Chungbuk National University Hospital (IRB No. 2021-10-040). Informed consent was not required since the medical data were to be used for research and anonymous analysis that is in line with the regulations of the Department of Health.

### 2.2. Study Population

**Figure 1:**
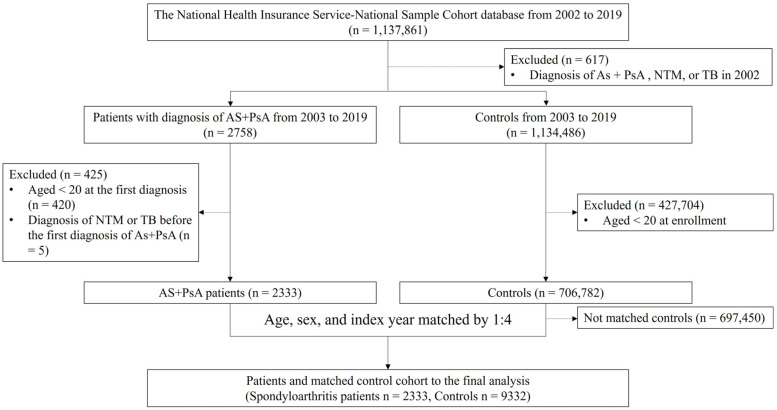
Patient selection flow diagram. Selection of patients with AS and PsA and matched controls was performed. AS, ankylosing spondylitis (International Classification of Diseases Tenth Revision [ICD-10]: M45); PsA, psoriatic arthritis (ICD-10: L40.5, M07.0-07.3, M09.00-M09.09); NTM, nontuberculous mycobacteria (ICD-10: A31); TB, tuberculous (ICD-10: A15-19, and National Health Insurance Service codes: V206, V246, and V000).

A flowchart of the data selection and classification is shown in Figure 1. The eligible patient group for the study was defined as individuals with AS (M45) and PsA (L40.5, M07.0-07.3, M09.00-M09.09), aged ≥20 years, and without prior diagnosis of NTM or MTB for at least 12 months before the first index date. Patients diagnosed with NTM or MTB during the 12-month pre-index period were excluded. Propensity score matching was used to select the control group based on age, sex, and index year.

### 2.3. Outcome Definition

The primary outcomes were the incidences and hazard ratios (HRs) of NTM and MTB. The secondary outcomes were the incidences of NTM or MTB according to the sex and age groups. In addition, the effects of biologic agents, including TNF-α inhibitors or IL-17 inhibitors, that were used by patients with SpA were also evaluated. NTM was analyzed using the ICD-10 code A31, and MTB was analyzed using the ICD-10 code A15-19 and the specific NHIS codes V206, V246, and V000.

### 2.4. Covariate Assessment

We assessed the variables potentially associated with MTB and NTM infections. These variables included age, sex, level of health insurance fees instead of household income (0–4, 5–6, 7–8, and 9–10), comorbidities (hypertension, diabetes, dyslipidemia, renal failure, ischemic heart disease, and cerebrovascular disease), smoking history (non-smoker, ex-smoker, and current smoker), body mass index (BMI), and medications for AS and PsA, which included TNF-α inhibitors and IL-17 inhibitors. Comorbidity was included in the analysis using the following ICD-10 codes: hypertension (I10–I15), diabetes (E10–E14), dyslipidemia (E78), renal failure (N17–N19), ischemic heart disease (I20–I25), and cerebrovascular disease (I60–I69). Drug use was defined as a prescription history for at least 30 days. Variables from the KNHIS-NCS database were included in the analysis.

Specifically, in the Cox proportional hazard model assessing risk factors for NTM and MTB in SpA, an incremental modeling approach was employed, resulting in the division into adjusted Models 1 and 2. This stepwise analysis aimed to confirm the influence of these variables. Adjusted Model 1 analyzed the HR of NTM and MTB after adjusting for variables such as sex, age group (20–29, 30–39, 40–49, 50–59, 60–69, 70–79, and ≥80), morbid obesity (body mass index ≥30.0), smoking status (non-smoker, ex-smoker, and current smoker), level of health insurance fees (0–4, 5–6, 7–8, and 9–10), and underlying comorbidities (hypertension, diabetes, dyslipidemia, renal failure, ischemic heart disease, and cerebrovascular disease). Adjusted Model 2 included the same variables as Model 1, incorporating the use of biological agents (TNF-α inhibitors or IL-17 inhibitors), and analyzed the HR of each NTM and MTB after adjustment.

### 2.5. Statistical Analysis

The baseline demographic and clinical characteristics of the patients with AS and PsA and controls were analyzed using the chi-square test. We compared the incidence rates of NTM and MTB in SpA patients and controls. Cox proportional hazard regression was used to evaluate the effect of biologics on SpA, NTM, and MTB. The results are presented as hazard ratios (HRs) and 95% confidence intervals (CIs). We used different adjustment models for potential confounders. Analyses were performed using SAS (version 9.4; SAS Institute Inc., Cary, NC) and R version 3.4.3 (R Foundation for Statistical Computing, Vienna, Austria). Statistical significance was set at *p* < 0.05.

## 3. Results

The general characteristics of the patients with SpA, including AS and PsA, and controls are presented in Table 1. All patients were equally distributed in terms of age and sex. The percentage of females was 49.5%. The distribution of age groups was similar among those in their 30s, 40s, 50s, and 60s. However, the percentage of individuals in their 20s was relatively low. AS is a disease commonly observed in individuals in their 20s and 30s. This result may be attributed to the inclusion of PsA, which exhibits heterogeneous characteristics. There was a statistically significant difference in smoking history between the control and SpA groups, with a notably higher percentage of ever-smokers in the control group. There was no economic disparity between the two groups when compared based on insurance level. The percentage of patients with hypertension, ischemic heart disease, and cerebrovascular disease was significantly higher in the SpA group. On the other hand, there were no differences in the prevalences of diabetes, dyslipidemia, and renal failure as comorbidities between SpA patients and the general population. Among patients with SpA, 9.6% were treated with TNF-α inhibitors and 0.04% were treated with IL-17 inhibitors.

Among the 2333 patients with SpA, only 4 (0.002%) developed NTM (Table 2) and 37 (0.016%) developed MTB (Table 3) after diagnosis with SpA. The cumulative incidence of NTM and MTB increased in the SpA group compared with that in the control group (Figure S1). In comparing the SpA group to the control group, no significant difference in the incidence rate ratio (IRR) was observed. A sub-analysis matched by sex and age group failed to reveal any statistically significant differences in NTM and MTB infection (Table 2 and Table 3). In patients with SpA, a sex disparity was observed in the incidence of NTM and MTB infections. Among the 4 patients with SpA who developed NTM, 3 were female, while among the 37 patients with SpA who developed MTB, 24 were male (Table 3).

In the case of NTM, the incidence was so low that it was difficult to attribute significance to the analysis by age group (Table 2). However, for MTB, although the incidence was low, differences could be identified by age group. In both the SpA and control groups, the incidence of MTB was highest in the 60s age group, exceeding that of individuals in their 40s and 50s by more than twofold. Specifically, among individuals in the SpA group, there were 22 MTB cases in patients aged 60 or older compared to 15 cases in patients aged under 60. In the control group, there were 64 individuals with MTB aged 60 or older compared to 35 individuals aged under 60 (Table 3).

Table 4 presents the comparison of the IRRs for NTM and MTB between patients with SpA treated with biologics compared to the control group. The IRRs for NTM and MTB were 5.66 (95% CI: 0.269, 16.752) and 3.069 (95% CI: 0.714, 3.708), respectively, which were not statistically significant. No cases of NTM or MTB were reported in female patients with SpA treated with biologics. In the case of SpA treated with biologics, it is challenging to attribute significance to NTM because there was only one reported case. Regarding MTB, only six cases (IR 3.863) were reported among male patients, and the incidence itself was low, making accurate evaluation difficult. However, there was a tendency for the incidence rate to increase compared to the control group, which had 48 cases (IR 0.997), although this difference was not statistically significant.

In the analysis of the Cox proportional HR among SpA patients, after adjusting for age, sex, smoking history, health insurance, and comorbidities, the adjusted HR for MTB was 1.479 (95% CI: 1.013, 2.158; *p* = 0.043), indicating a significant increase (Table 5, Model 1). However, upon further adjustment for the influence of biological therapy (Table 5, Model 2), this association was not statistically significant.

## 4. Discussion

The present study aimed to compare the incidences of NTM and MTB in the SpA cohort with those in the control group, and to determine whether biologic agents affect the development of NTM and MTB. The analysis revealed that the IRRs for both NTM and MTB did not increase compared to that of the general population, and this remained unchanged regardless of biologic therapy use.

In a population-based study conducted in Brazil, a country with a high incidence of TB, researchers reported an increased risk of TB associated with the use of TNF-α inhibitors in patients with rheumatic diseases. One study found that therapy with TNF inhibitors significantly increased the number of TB cases among patients with systemic rheumatic diseases [14]. However, several studies have reported that TNF-α inhibitors or IL-17 inhibitors do not increase the risk of active TB [15,16,17,18]. In a previous Korean nationwide nested case-control analysis that was conducted to assess the risk of cancer, TB, and serious infections in patients with AS, PsA, and psoriasis treated with IL-17 and TNF-α inhibitors, it was shown that IL-17 inhibitors may offer an advantage in terms of TB risk [19].

Our study did not show a significant increase in the IRRs of NTM and MTB infections, even with the use of biologics, in patients with SpA. In addition, a sub-analysis matched by sex and age group also failed to reveal any statistically significant differences in NTM and MTB infections. However, in patients with SpA, a sex disparity was observed in the incidence of NTM and MTB infections. Among the 4 patients with SpA who developed NTM, 3 were female (75%), while among the 37 patients with SpA who developed MTB, 24 (64.8%) were male.

In our study, although the total number of MTB patients was small, in both the SpA patient group and the control group the incidence of MTB was higher in individuals over 60 years old compared to those under 60 years old. This suggests that individuals over 60 years old should be particularly cautious regarding the risk of MTB. Furthermore, this result emphasizes the importance of differentiating latent TB and performing prophylaxis when biologics are used in SpA patients, especially in this older age group.

In some results of the Cox proportional HR analysis, the HR for MTB was statistically significant. This significance was observed in the unadjusted HR, which was only adjusted for age and sex, and in the results adjusted for BMI, smoking status, insurance level, and underlying comorbidities. The HR was 1.5 times higher in patients with SpA than in the general population. In Model 2, which was adjusted for the use of biologics, the association was not statistically significant. This suggests that biological therapy in patients with SpA may be a risk factor for MTB infection.

This study had several limitations. First, questionnaire data that can consider the disease activity of patients with SpA or blood test results that can confirm inflammation, such as ESR and CRP levels, were not obtained. Another limitation of our study was the lack of the acid-fast bacilli staining, culture, purified protein derivative tests, or interferon-gamma release assays. However, it is important to note that NTM and MTB diagnoses by ICD code are considered reliable because they are only recorded when supported by microbiological evidence. In addition, our data contained data from before the rare intractable diseases (RID) system, wherein the Korean government provided financial support to patients with rare incurable rheumatic diseases, including AS and PsA, which have been specially registered and managed since 2009. To be eligible for registration in the RID system, patients must meet the diagnostic criteria for each RID and be carefully reviewed by the corresponding healthcare institution. Although our data included data from 2002 before the RID system, rheumatic diseases such AS and PsA were directly registered with ICD codes after careful review by rheumatologists; therefore, the analysis using diagnostic codes is reliable. Finally, our study encountered challenges owing to its focus on low-incidence diseases, such as NTM, and its target patient group, who were individuals with SpA, which is also a rare condition. The limited sample size presented difficulties with our analysis. Despite these challenges, the investigation of MTB infections in patients with SpA, especially in the context of biological therapy, addresses critical public health issues. We believe that the findings of our study are of considerable significance in addressing these concerns.

## 5. Conclusions

Our study showed that the risks of NTM and MTB infection did not increase in patients with SpA. However, some of our study results seem to be affected by biologics, implying that biologics may serve as risk factors for MTB infections in patients with SpA. Therefore, we suggest that proactive screening for latent TB infection and administration of adequate prophylaxis when using biologics can mitigate the risk of MTB infection. Additionally, this point should be further emphasized in the older age group for patients over 60 years old.

## Figures and Tables

**Table 1:** Baseline characteristics of the study cohort and controls.

Variables	Spondyloarthritis Patients	Controls	*p*-Value
N	2333	9332	
Female, N (%)	1155 (49.51)	4620 (49.51)	>0.999
Age, years, N (%)			>0.999
20–29	164 (7.03)	656 (7.03)	
30–39	400 (17.15)	1600 (17.15)	
40–49	425 (18.22)	1700 (18.22)	
50–59	415 (17.79)	1660 (17.79)	
60–69	461 (19.76)	1844 (19.76)	
70–79	355 (15.22)	1420 (15.22)	
≥80	113 (4.84)	452 (4.84)	
Morbidly obese (BMI ≥30.0), N (%)	31 (1.33)	108 (1.16)	0.728
Ever-smoker, N (%)	616 (26.40)	2501 (26.80)	<0.0001
Level of health insurance fees, N (%)			0.577
0–4	803 (34.42)	3232 (34.63)	
5–6	413 (17.70)	1617 (17.33)	
7–8	501 (21.47)	2111 (22.62)	
9–10	616 (26.40)	2372 (25.42)	
Comorbidity (ICD-10 code), N (%)			
Hypertension (I10–I15)	898 (38.49)	3137 (33.62)	<0.0001
Diabetes (E10–E14)	212 (9.09)	775 (8.30)	0.241
Dyslipidemia (E78)	9 (0.39)	35 (0.38)	0.999
Renal failure (N17–N19)	15 (0.64)	73 (0.78)	0.574
Ischemic heart disease (I20–I25)	78 (3.34)	194 (2.08)	<0.0001
Cerebrovascular disease (I60–I69)	88 (3.77)	259 (2.78)	0.014
Use of biological agents, N (%)			<0.0001
TNF-α inhibitors	224 (9.60)	2 (0.02)	
IL-17 inhibitors	1 (0.04)	0 (0.00)	

The *p*-value was estimated using the chi-square test. BMI, body mass index; ICD-10, International Classification of Diseases Tenth Revision; TNF, tumor necrosis factor; IL, interleukin.

**Table 2:** Incidence of NTM in patients with SpA compared to controls stratified by sex or age.

Variables	SpA (N = 2333)			Controls (N = 9332)			IRR (95% CI) of NTM
	N (%)	PY	IR	N (%)	PY	IR	
Overall	4 (0.002)	24.837	0.161	9 (0.001)	100.078	0.090	1.789 (0.396, 4.180)
Male	1 (0.000)	12.021	0.083	3 (0.000)	48.305	0.062	1.339 (0.118, 10.912)
Female	3 (0.001)	12.825	0.234	6 (0.001)	51.773	0.116	2.017 (0.339, 5.423)
Age, years							
20–29	0 (0.000)	2.274	0.000	0 (0.000)	9.180	0.000	-
30–39	0 (0.000)	4.385	0.000	0 (0.000)	17.511	0.000	-
40–49	1 (0.000)	4.420	0.226	2 (0.000)	17.600	0.114	1.982 (0.122, 14.846)
50–59	2 (0.001)	4.278	0.468	1 (0.000)	17.156	0.058	8.069 (0.225, 27.312)
60–69	1 (0.000)	5.304	0.189	4 (0.000)	21.215	0.189	1.000 (0.112, 8.947)
70–79	0 (0.000)	3.404	0.000	2 (0.000)	14.174	0.141	-
≥80	0 (0.000)	0.772	0.000	0 (0.000)	3.242	0.000	-

NTM, nontuberculous mycobacteria; SpA, spondyloarthritis; PY, 1000 person-years; IR, incidence rate; IRR, incidence rate ratio; CI, confidence intervals.

**Table 3:** Incidence of MTB in patients with SpA compared to controls stratified by sex or age.

Variables	SpA (N = 2333)			Controls (N = 9332)			IRR (95% CI) of MTB
	N (%)	PY	IR	N (%)	PY	IR	
Overall	37 (0.016)	24.664	1.500	101 (0.011)	99.727	1.013	1.481 (0.814, 1.728)
Male	24 (0.010)	11.888	2.019	48 (0.005)	48.143	0.997	2.025 (0.832, 2.218)
Female	13 (0.006)	12.776	1.018	53 (0.006)	51.584	1.027	0.991 (0.543, 1.827)
Age, years							
20–29	2 (0.001)	2.262	0.884	2 (0.000)	9.176	0.218	4.055 (0.259, 13.040)
30–39	3 (0.001)	4.360	0.688	4 (0.000)	17.486	0.229	3.004 (0.361, 7.205)
40–49	3 (0.001)	4.401	0.682	14 (0.002)	17.529	0.799	0.854 (0.268, 3.249)
50–59	7 (0.003)	4.238	1.652	15 (0.002)	17.081	0.878	1.882 (0.537, 3.227)
60–69	13 (0.006)	5.258	2.472	33 (0.004)	21.120	1.563	1.582 (0.642, 2.318)
70–79	8 (0.003)	3.382	2.365	25 (0.003)	14.120	1.771	1.335 (0.511, 2.514)
≥80	1 (0.000)	0.763	1.311	8 (0.001)	3.215	2.488	0.527 (0.095, 6.054)

MTB, Mycobacterium tuberculosis; SpA, spondyloarthritis; PY, 1000 person-years; IR, incidence rate; IRR, incidence rate ratio; CI, confidence intervals.

**Table 4:** Incidence of NTM and MTB between patients with SpA treated with biologics compared to controls, stratified by sex.

Variables	SpA Treated with Biologics			Controls (N = 9332)			IRR (95% CI)
	N (%)	PY	IR	N (%)	PY	IR	
NTM							
Overall	1 (0.004)	1.965	0.509	9 (0.001)	100.078	0.090	5.656 (0.269, 16.752)
Male	1 (0.004)	1.588	0.630	3 (0.000)	48.305	0.062	10.161 (0.285, 26.316)
Female	0 (0.000)	0.377	0.000	6 (0.001)	51.773	0.116	-
MTB							
Overall	6 (0.027)	1.930	3.109	101 (0.011)	99.727	1.013	3.069 (0.714, 3.708)
Male	6 (0.027)	1.553	3.863	48 (0.005)	48.143	0.997	3.875 (0.771, 4.208)
Female	0 (0.000)	0.377	0.0 00	53 (0.006)	51.584	1.027	-

NTM, nontuberculous mycobacteria; MTB, Mycobacterium tuberculosis; SpA, spondyloarthritis; PY, person-years; IR, incidence rate; IRR, incidence rate ratio; CI, confidence intervals.

**Table 5:** Cox proportional hazard model of risk factors for NTM and MTB in SpA.

Outcomes	No. of Events		Unadjusted HR (95% CI)	*p*-Value	Adjusted Model 1 HR (95% CI) ^1^	*p*-Value	Adjusted Model 2 HR (95% CI) ^2^	*p*-Value
	SpA (N = 2333, 24,650 PY)	Control (N = 9332, 99,694 PY)						
NTM	4	9	1.793 (0.552, 5.821)	0.332	1.753 (0.538, 5.712)	0.352	1.373 (0.378, 4.989)	0.630
MTB	37	101	1.482 (1.017, 2.160)	0.041 *	1.479 (1.013, 2.158)	0.043 *	1.303 (0.872, 1.947)	0.196

^1^ Adjusted Model 1: sex, age group (20–29, 30–39, 40–49, 50–59, 60–69, 70–79, and ≥80), morbidly obese (body mass index ≥30.0), smoking status (non-smoker, ex-smoker, and current smoker), level of health insurance fees (0–4, 5–6, 7–8, and 9–10), and underlying comorbidity (hypertension, diabetes, dyslipidemia, renal failure, ischemic heart disease, and cerebrovascular disease). ^2^ Adjusted Model 2: sex, age group (20–29, 30–39, 40–49, 50–59, 60–69, 70–79, and ≥80), morbidly obese (body mass index ≥30.0), smoking status (non-smoker, ex-smoker, and current smoker), level of health insurance fees (0–4, 5–6, 7–8, and 9–10), underlying comorbidity (hypertension, diabetes, dyslipidemia, renal failure, ischemic heart disease, and cerebrovascular disease), and use of biological agents (TNF-α inhibitors or IL-17 inhibitors). * *p*-value < 0.05. NTM, nontuberculous mycobacteria; MTB, Mycobacterium tuberculosis; SpA, spondyloarthritis; PY, person-years; HR, hazard ratio; CI, confidence intervals; TNF, tumor necrosis factor; IL, interleukin.

## Data Availability

Data cannot be shared publicly because they belong to the National Health Insurance Service (NHIS). There are ethical restrictions on sharing such a dataset because these data contain potentially identifying or sensitive patient information. To request data from NHIS, researchers have to apply during the recruitment period and submit a research proposal. Raw data are available to researchers upon reasonable academic request and with the permission of the Korean NHIS Institutional Data Access (https://nhiss.nhis.or.kr/bd/af/bdafa001lv.do, accessed on 24 May 2022). The authors had no special access privileges.

## Supplementary Materials

The following supporting information can be downloaded at: https://www.mdpi.com/article/10.3390/medicina60040579/s1, Figure S1: Cumulative incidence of (a) MTB and (b) NTM in patients with SpA.
