# Supplementary material for: Tuberculosis and Nontuberculous Mycobacterial Infections in Patients with Spondyloarthritis: A Population-Based Study
Source: Medicina (Kaunas). 2024 Mar 31;60(4):579. doi: 10.3390/medicina60040579 (PMC11052061; doi:10.3390/medicina60040579)
Supplement: Supplementary file 1 [file medicina-60-00579-s001.zip › medicina-2888417-supplementary.pdf]

Figure S1. Cumulative incidence of (a) MTB and (b) NTM in patients with SpA.

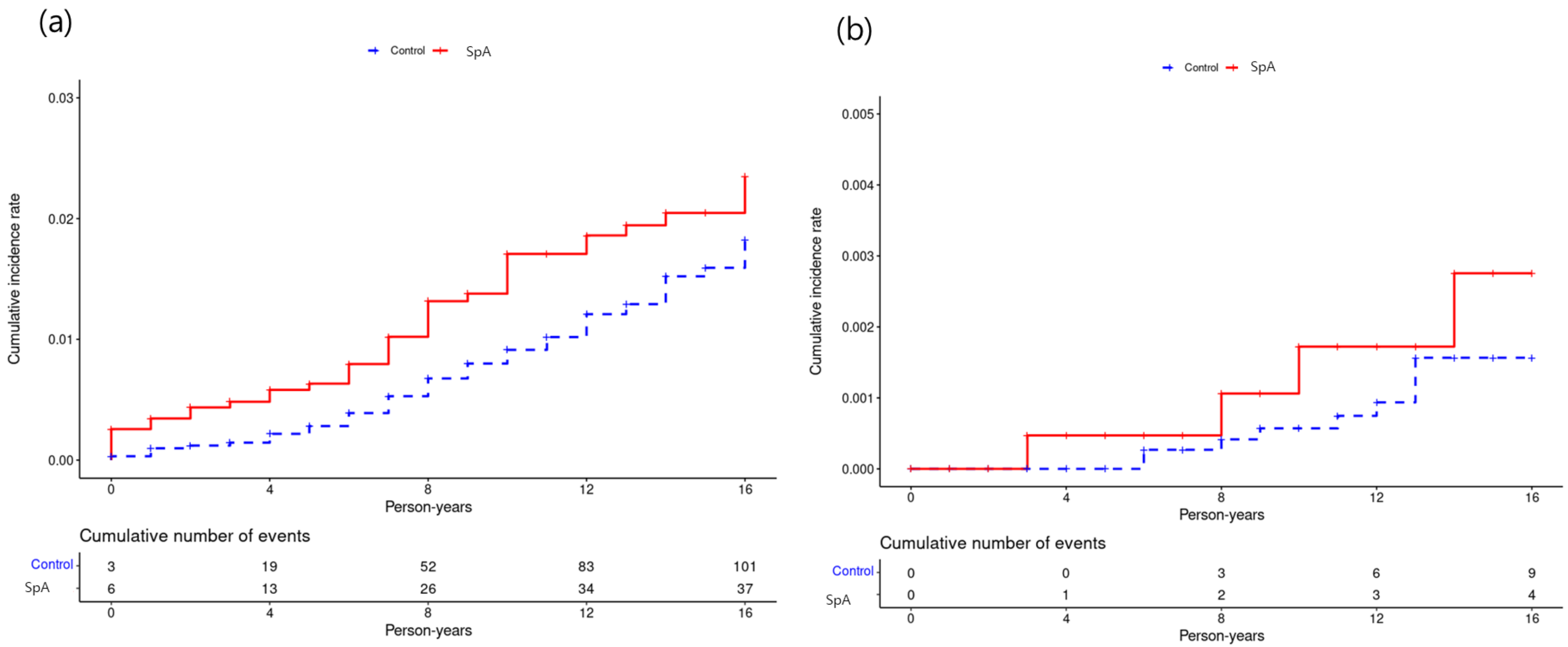

SpA, spondyloarthritis; MTB, mycobacterium tuberculosis; NTM, nontuberculous mycobacteria.
